# Treatment of Sub-Acute and Chronic Gout

**Published:** 1880-04

**Authors:** Alexander Hadden


### TREATMENT OF SUBACUTE AND CHRONIC GOUT.

BY ALEXANDER HADDEN, M.D.

Gout is an affection which seems to be as old as civilization itself. Nearly all the physicians of the early periods of medical science, who have left any records, have made mention of it. The description of it given by Hippocrates, Galen, Celsus, and others of their respective times, show clearly that they understood the disease as well as did those of a later date, as Sydenham, Wollaston, Boerhaave, etc., and with the exception of the contributions made by modern chemistry and microscopy, their knowledge of the subject was fully equal to that of the physicians of our own time. Among the most prominent of these last who have written upon the subject and whose names are familiar to the profession, may be mentioned Garrod, Todd, Watson, and Budd.

By universal consent the presence of uric acid in the blood and urine, where it exists in the form of urate of soda, and the deposit of the same in the joints, has now become a diagnostic sign, one which pre-eminently distinguishes gout from all diseases of a kindred nature. I will therefore make direct reference to this fact as a cardinal point in the treatment of the malady. Certain circumstances, together with the treatment of a number of cases, with well-demonstrated results, have lately shown to my mind conclusively that some things usually set forth as the prominent causes of gout, are only apparently such, and this, because of their intimate association with the real cause; I refer now to a diet embracing a large proportion of flesh meats and other articles containing a large amount of the nitrogenous and albuminous principles.

Heretofore, all authors who have written upon gout have united in classing foods of the character named as among the chief factors in the production of this disease; hence on their treatment of it such have been, to a great extent, excluded from the dietary of the patient. Now, free livers, as a general rule, are large consumers of animal food, as well as of wine, beer, etc., and leading at the same time, perhaps, inactive lives. Whether it was the association of the meat diet with these other conditions that led to this conclusion, thus attributing to one cause what was in reality due to another, I cannot say; but the fact is as stated.

Dr. Garrod, in his remarks upon the causes of gout, seems to be somewhat in doubt when speaking of the effects of various kinds of food upon different animals. He instances the tortoise, feeding exclusively upon lettuce, and excreting the urates in greater proportion than the dog which feeds upon animal food. Sir Thomas Watson, also, is not quite clear when treating of the causes of gout. He mentions the case of butchers, who are rarely afflicted with the disease, though, as might be expected, great consumers of the commodity in which they deal. Men and women of sedentary habits, according to him, are not especially apt to suffer from gout, while, on the other hand, the huntsman, a free liver, but taking an abundance of exercise in the open air, does so suffer.

Dr. Budd seems to be more positive in his opinions as to the causes of gout. He speaks of the ballasters of the Thames who drink excessively of malt liquors, and who almost universally suffer from this malady, although taking a large amount of out-door exercise.

Dr. H. Bentley Todd, in an article on gout in the London *Journal of the Medical Sciences* for December, 1843, recommends, in a very cautious way, the employment of a moderate amount of animal food in the treatment of this disease, discarding, at the same time, all vegetables having saccharine properties, or which might be prone to acetous fermentation in the stomach and duodenum, and thinks that a diet of this kind, embracing a fair proportion of animal food, and regulated as to quantity, would be the one most conducive to the establishment of a healthy digestion. This author, I may here remark, is the only one, so far as my reading extends, who does not assign flesh food as a cause of gout, and show a disposition to exclude it from the bill of fare in the treatment of this disease.

In regard to the different theories that have been from time to time enunciated by medical men in regard to the pathology of gout, they are too numerous to dwell upon here; but the cases which I shall present will bring out some new facts, or at least some facts not hitherto observed, in the treatment of this malady.

Case I.—D. B. P——, æt. 50; native of the U. S.; married; by profession, lawyer; residence, Brooklyn, N. Y.; came into my service at the Presbyterian Hospital Dec. 1, 1879, having been a helpless sufferer from gout for eighteen months previously. He had been, when in health, a man of fine physique, tough and rugged, descended from a long-lived ancestry, both parents dying at advanced ages, and giving no hereditary history of the affection in question. He had been a free liver, and though not intemperate, had yet indulged freely in almost all kinds of alcoholic beverages. When he came under my care his condition was as follows: Feet and hands very much swollen, especially the joints of the fingers, which were also deformed by gouty deposits; the joints of the toes the same; the knees, shoulders and elbows also swollen, so that he was unable to move either arms or hands. Almost every joint in the body was stiff and painful; even the temporo-maxillary articulations were stiff and painful to the degree that he could not open his mouth sufficiently to take food, except such as had been reduced to a fluid or semi-fluid state. He was confined to bed and unable to move even a finger; was in fact totally helpless, and had, besides, severe gouty conjunctivitis. The heart's action was feeble, but no organic change had yet taken place. Kidneys healthy; no evidence of albumen or casts. Large quantities of the urates of soda were eliminated by these organs. Lungs large and healthy, and brain clear. He suffered at times from intestinal inflatus, and at times from diarrhœa, alternating with constipation. It was, in fine, a typical case of an advanced stage of gout. The patient had been subjected to all the ordinary and well-known methods of treatment usually employed in such cases, but without anything more than very temporary relief. The disease was thought to be in a progressive state, and the case regarded as hopeless.

On December 8, 1879, patient was put upon the following treatment: a diet consisting almost wholly of animal and albuminous foods, with such vegetables as contained neither amylaceous nor saccharine principles, such as white turnip, spinach, leeks, celery, raw cabbage, onions, both raw and cooked, potatoes sliced thin, fried and crisp, gluten bread toasted. All stimulants, both alcoholic and malt liquors, were withheld. When diarrhœa was present a sufficient amount of opium was given to control it. The exhibition of salicylicate of soda, which the patient had been taking previously, was continued in small doses several times a day, so far as to relieve any occasional rise of temperature, and check any fermentation of food that might take place, but not with a view to cure the disease. The wrists and hands were encased in plaster-of-Paris, so as to prevent all muscular movements. Under this treatment the patient began slowly to improve. The urates disappeared from his urine, he became free from pain, was soon able to sit up the greater part of the day, and finally he became able to walk. At this writing, March 15, improvement still coutinues.

Case II.—Rev. Charles P——, æt. 57; native of the United States; married; by profession a teacher; was a case of hereditary gout, several of the patient's ancestors and near relatives having suffered from the disease. Since 1848 he has himself been subject to attacks of the malady, which were sometimes attended with great cerebral disturbance, causing him to lie upon one occasion for three weeks unconscious. These attacks became more frequent and of longer duration, until in the autumn of 1875 he became a confirmed invalid, and has so remained up to the time of his coming under my care. He would be sometimes, as he himself states, bright and cheerful; but most of the time he was unable to attend to the duties of his profession, or even to walk. In the spring of 1879 he experienced some mitigation of his sufferings; but in September of that year, although residing in a district of country famous for its salubrity, he was taken down with chills and fever, which continued for three weeks, and from this time he never left his bed until he set out for the Presbyterian Hospital, New York, where he arrived December 6th, and I saw him for the first time on the 7th.

In this case, as in No. 1, nearly all the joints of the body, both large and small, were inflamed, swollen and painful. He had lost all appetite, food become distasteful; his digestion was much disordered; there was borborygmus, great and frequent evolutions of gas, and the bowels were obstinately constipated. The circulation was feeble, 42–45, and intermittent. He had been rapidly losing flesh and strength, and the condition of his nervous system was such that he could endure neither light nor sound. He had been living mostly upon farinaceous food, fruits and milk. He had taken all the remedies usually prescribed in such cases, and also stimulants, both spirituous and malt, but without benefit.

He was first ordered a cathartic pill of compcolocynth, in order to clear his bowels. He was then put upon salicylic acid and ordered to take hyd. chloral gr. x., potass. brom. gr. xx., at bed time, to make him sleep. On December 8th he was put upon the meat and non-amylaceous and non-saccharine diet, with a bill of fare substantially the same as that ordered for case No. 1. On this treatment, by January 10, 1880, one month from its commencement, the urine of the patient, which had been at first acid, now became neutral, and the urates had wholly disappeared. By the 18th, the urine showed an alkaline reaction. The deposits in the joints were now rapidly disappearing, the patient was free from pain and going about the hospital, and on fine days going outside to walk. Improvements still continued up to 1st March, when he left the hospital on a visit to his friends.

Case III.—S. J——, æt. 22; native of the United States; single; conductor on horse-cars. Two years ago had an attack of gout in right foot, which continued for two months, and was followed by pain and swelling in the right knee and hip. Both hands were also slightly swollen and stiff. No distortion, however, had yet taken place in any of the joints. These conditions continued with intermissions up to the time of his admission. On January 8th, he was put upon the meat and non-amylaceous and non-saccharine diet, and ordered to take salicylate of soda. Improvement in patient's condition was soon manifest, the urates disappeared from his urine, his hands became quite supple; but as he was discharged for insubordination on the 26th, having been in the hospital only a little over three weeks, the time was too short to show the full effects of the treatment; but so far as the case had progressed, the same favorable results were seen as in the preceding cases.

Case IV.—M. G——, æt. 35, native of Ireland; married; by occupation housekeeper. Has had trouble with her feet for the last five years, interfering very materially with locomotion. About six weeks previous to admission her feet became œdematous and the ankle joints very tender. The knees became involved subsequently, and finally the wrist and elbow. All these joints were swollen and tender, but never red. At the same time she was taken sick with flatulence and pain at the epigastrium; symptoms all pointing plainly to a case of gout.

On December 6th, she was ordered to be put on the flesh and non-amylaceous and non saccharine diet, and the right hand to be put in a plaster-of-Paris bandage. In this case, as in the others, the urates disappeared from the urine, and the specific gravity steadily increased from 1012 1015, then to 1018, and finally to 1020, and the patient's general health showed manifest improvement. On the 26th, the diet was again changed to one purely vegetable. Although the effect was not at once seen in the urine, it was immediately apparent in the general health of the patient. The tone of the system was evidently lowered. But on the 5th of the following month, twelve days after the change to a vegetable diet had been made, the urates reappeared in the urine, when she was again put upon the animal diet. The patient left the hospital a few days afterward at her own request; so that I have no means of knowing the final result. The history of the case is given in this connection, simply because it illustrates so well the effect of the meat and albuminous diet in causing the urates to disappear from the urine; and then again the result from the employment of a vegetable diet in causing their reappearance in large quantities.

I would call especial attention to this method of putting gouty patients upon a diet of strictly animal and albuminous foods, together with such vegetables as are destitute of amylaceous and saccharine properties; for that is the central fact in the above plan of treatment. It may be noticed, too, that in the treatment of these cases none of the usual medical agents have been employed, with the exception of salicylate of soda, and such others as were from time to time indicated for the correction of merely temporary conditions.

As has already been shown, gout may be produced under many and various conditions and circumstances: either where there is a hereditary predisposition to the malady, or without; under circumstances of active exertion, or where persons have led sedentary lives; where there has been use and even abuse of the stronger alcoholic beverages, or where the subject has been strictly temperate, even abstinent. Under each or all of the above conditions, persons may have the gout or escape it entirely. But while this is the case, it is a settled fact that those who live largely upon animal food are less liable to the disease than others; while, according to Dr. Gubler of Paris, who has recently been making extensive researches into diseases of the blood-vessels, those living upon an exclusively vegetable diet, as do many of the French peasantry, and more notably the monks of La Trappe, are peculiarly subject to chalky or calcareous deposits in the arteries. The same is true in animals and birds as well. Siedamgrotzski states that gouty deposites take place in the pigeon; also in the turkey under domestication, and in the common barn-yard fowl. In further confirmation of this we have the testimony of Mr. Wm. A. Conklin, Director of the Zoological Garden in Central Park, New York, who, in reply to my inquiry, affirms that it is only the herbivorous and granivorous animals and birds that are subject to such deposits, while the carnivora escape them entirely. This fact alone might, I should think, indicate the point of departure in the treatment of this disease.

Might it not be, too, that in the case of animals and birds, as also in the case of the human subject, feeding upon too exclusive a diet of vegetable food, abounding in amylaceous and saccharine elements, under certain conditions a species of fermentation is set up, resulting in certain chemical changes somewhat analagous to the fermentation of vegetable matter out of the body, whereby certain gases are evolved and certain properties generated? For example, the fermentation by which wine and beer are produed.

